# Diltiazem inhibits breast cancer metastasis via mediating growth differentiation factor 15 and epithelial-mesenchymal transition

**DOI:** 10.1038/s41389-022-00423-5

**Published:** 2022-08-13

**Authors:** Yen-Chang Chen, Chen-Teng Wu, Jia-Hong Chen, Cheng-Fang Tsai, Chen-Yun Wu, Pei-Chun Chang, Wei-Lan Yeh

**Affiliations:** 1 Institute of New Drug Development, China Medical University, No.91 Hsueh-Shih Road, Taichung, 404333 Taiwan; 2 Department of Surgery, China Medical University Hospital, No. 2, Yude Road, Taichung, 404332 Taiwan; 3 Department of General Surgery, Taichung Tzu Chi Hospital, Buddhist Tzu Chi Medical Foundation, No. 88, Sec. 1, Fengxing Road, Taichung, 427213 Taiwan; 4 Department of Medical Laboratory Science and Biotechnology, Asia University, No.500 Lioufeng Road, Taichung, 413305 Taiwan; 5 Department of Bioinformatics and Medical Engineering, Asia University, No.500 Lioufeng Road, Taichung, 413305 Taiwan; 6 Department of Biochemistry, School of Medicine, China Medical University, No.91 Hsueh-Shih Road, Taichung, 404333 Taiwan

**Keywords:** Breast cancer, Target identification

## Abstract

Migration and metastasis commonly happen to triple-negative breast cancer (TNBC) patients with advanced diseases. In many studies, it has been suggested that epithelial-mesenchymal transition (EMT) is one of the key mechanisms triggering cancer metastasis. Accumulating evidence has proven that calcium channel blockers mediate cell motility. Therefore, we attempt to investigate the effects of diltiazem, which has been selected from several FDA-approved clinical calcium channel blockers, on EMT in TNBC. By using both mouse and human TNBC cell lines, we found that diltiazem decreases colony formation and cell migration in breast cancer cells. The expression of epithelial markers such as E-cadherin and ZO-1 were increased dose-dependently by diltiazem, while mesenchymal markers such as Snail and Twist were decreased. In addition, we found that the expression of growth differentiation factor-15 (GDF-15) was also increased by diltiazem. Administering recombinant GDF-15 also reverses EMT, inhibits colony formation and migration in breast cancer cells. Moreover, treatment with diltiazem in tumor-bearing mice also decreases cancer metastasis and nodule formation, with more GDF-15 expression in diltiazem-treated mice than saline-treated mice, respectively. These findings suggest that diltiazem regulates EMT and cell motility through elevating GDF-15 expression in breast cancers in vitro and in vivo.

## Introduction

Metastasis is a common and complex process in advanced cancer stages and it causes fatal risk in most cancer patients [1, 2]. In late-stage breast cancer patients, approximately 60% of the primary tumors recur as well as transfer to lung or bone [3, 4]. This makes both intractable treatment and high rate of mortality. Previous studies have implicated that triple-negative or basal-like breast cancer patients experience reduced overall and disease-free survival compared with patients with other breast cancer subtypes [5, 6], partially because of the higher local recurrence and distant metastasis rates [7].

Epithelial-mesenchymal transition (EMT) is a crucial process involved in cancer metastasis [8]. Through upregulating the expression of mesenchymal-related genes, epithelial cells disassemble the polarity and cell-cell junctions in order to migrate and invade [9]. In addition, zinc-finger-family of transcription factors such as Twist, Snail, and matrix metalloproteinases (MMPs) are upregulated during EMT, leading to aggressive cancer migration, invasion and cell growth [10–12].

Growth differentiation factor-15 (GDF-15), also known as macrophage inhibitory cytokine (MIC)-1 or nonsteroidal anti-inflammatory drug-activated gene (NAG)-1, is a member of TGF-β superfamily [13]. It has been reported to involve in cardio and renal protection, cellular stress responses and metabolic related diseases [13–15]. However, the role of GDF-15 is disputed among cancers since it involves in tumor behaviors both positively and negatively depending on the cellular state and environment [16]. The altered expression of GDF-15 influence proliferation, metastasis, immune escape, and drug resistance [17, 18]. The functions and regulatory mechanisms of GDF-15 are controversial and worth further investigation.

Diltiazem, an FDA-approved antihypertensive drug, is an L-type voltage-gated calcium channel blocker (CCB) [19]. Previous studies revealed the anti-tumor effects of some CCBs among different cancer types, including induction of autophagy and apoptosis [20], inhibition of cancer growth [21], or reverses chemotherapeutic-resistance through inhibiting multidrug resistance protein 1 [22, 23]. Evidence that suggests the anti-tumor effect of CCBs is accumulating, however, the influences of diltiazem are still vague. In this presented study, we addresses the anti-tumor effects of diltiazem which inhibits cell motility and EMT through elevating GDF-15 expression in triple-negative breast cancer in vitro and in vivo.

## Results

### Diltiazem decreases cell motility and epithelial-mesenchymal transition in breast cancer cells

**Fig. 1:**
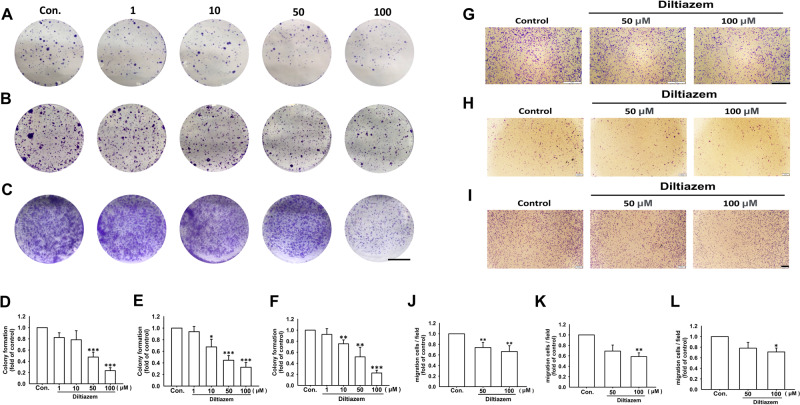
Colony formation and cell migration are inhibited by diltiazem in breast cancer cell lines. After diltiazem treatment (1–100 μM) for 10 days, the number of colonies formed (bigger than 1 mm in diameter) were counted. Note that colony formation of JC (**A**, **D**), 4T1 (**B**, **E**), and MDA-MB-231 cells (**C**, **F**) were all inhibited by diltiazem. Scale bar, 1 cm. In cell culture insert system, diltiazem (50 or 100 μM) treatment for 24 h also reduced cell migration in JC (**G**, **J**), 4T1 (**H**, **K**), and MDA-MB-231 cells (**I**, **L**). Scale bar, 400 μm. Graphs showed mean ± S.D. of three independent experiments. *p* value was calculated using Student’s t test. *p < 0.05; **p < 0.01; ***p < 0.001 compared to control group.

Appropriate diltiazem concentration was chosen according to MTT assay and proliferation examination which did not cause marked cell death under indicated treatment (Supplementary Fig. 1). After treating diltiazem for 10 days, colonies formed of JC (Fig. 1A, D), 4T1 (Fig. 1B, E) and MDA-MB-231 cells (Fig. 1C, F) were significantly diminished in a dose-dependent manner. Colony formation was reduced to 0.23 ± 0.05-fold, 0.33 ± 0.08-fold, and 0.22 ± 0.05-fold of control under 100 μM diltiazem treatment on JC, 4T1, and MDA-MB-231 cells, respectively. In cell culture insert system, treatment of diltiazem for 24 h markedly decreased cell migration through transwell membrane compared with vehicle-treated control group in JC (Fig. 1G, J), 4T1 (Fig. 1H, K) and MDA-MB-231 cells (Fig. 1I, L). Cell migration was reduced to 0.66 ± 0.11-fold, 0.59 ± 0.12-fold, and 0.63 ± 0.02-fold of control under 100 μM diltiazem treatment on JC, 4T1, and MDA-MB-231 cells, respectively. These findings suggest that colony formation and cell migration ability were inhibited by diltiazem in triple-negative breast cancer cells. Moreover, the invasive motility of breast cancer cells was also elucidated. In Supplementary Fig. 2, the invasive ability and would healing ability were both significantly decreased by diltiazem in 4T1 and MDA-MB-231 cells in dose-dependent manners.

**Fig. 2:**
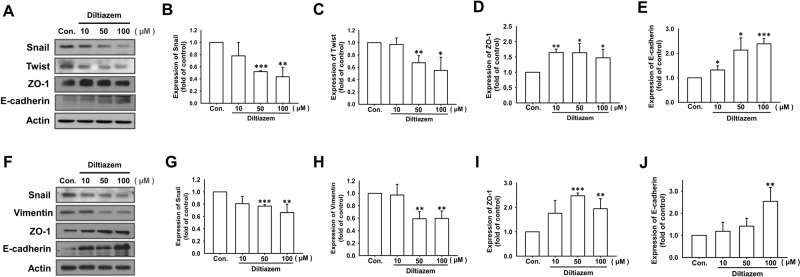
Diltiazem abrogates epithelial-mesenchymal transition by increasing epithelial markers expression and decreasing mesenchymal markers expression in breast cancer cell lines. Cells were treated with diltiazem (10–100 μM) for 24 h, and protein expressions were detected by Western blot. It is shown that diltiazem decreased the expression of mesenchymal markers (Snail, Twist and vimentin) but increased the expression of epithelial markers (E-cadherin and ZO-1) on 4T1 (**A**–**E**) and MDA-MB-231 (**F**–**J**) breast cancer cells. Graphs showed mean ± S.D. of three independent experiments. *p* value was calculated using Student’s *t* test. *p < 0.05; **p < 0.01; ***p < 0.001 compared to control group.

Next, we evaluated the effects of diltiazem on epithelial-mesenchymal transition, since EMT mediates one of the major migration mechanism of cancer cells. After treating by diltiazem for 24 h, protein expression of mesenchymal markers including Snail, Twist, and vimentin were decreased in both 4T1 (Fig. 2A–C) and MDA-MB-231 cells (Fig. 2F to H). On the other hand, protein expressions of zonula occludens-1 (ZO-1) and E-cadherin representing as epithelial markers were significantly elevated by diltiazem in both 4T1 (Fig. 2A, D, E) and MDA-MB-231 cells (Fig. 2F, I, J).

**Fig. 3:**
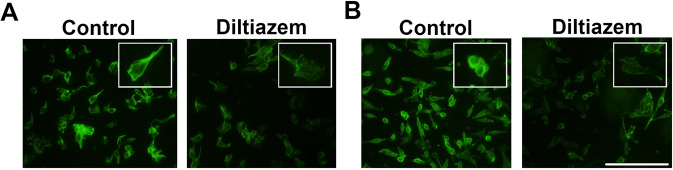
Diltiazem attenuates F-actin disassembly and reorganization in breast cancer cell lines. After treating with 50 μM diltiazem for 24 h, F-actin dynamics was revealed by FITC-phalloidin staining. Note that F-actin disassembling was attenuated by diltiazem on both 4T1 (**A**) and MDA-MB-231 cells (**B**) in representative images. Scale bar, 100 μm.

In addition, actin dynamics commonly occurs during epithelial-mesenchymal transition [24]. F-actin or stress fiber disassembles and rearranges to promote migration [25]. As demonstrated in Fig. 3, diltiazem remarkably antagonized F-actin disassembly shown by FITC-phalloidin staining in both 4T1 and MDA-MB-231 breast cancer cells. Taken together, these findings suggest that diltiazem reverses expression of EMT markers and may inhibit breast cancer motility.

### Diltiazem mediates GDF-15 expression in breast cancer cells

**Fig. 4:**
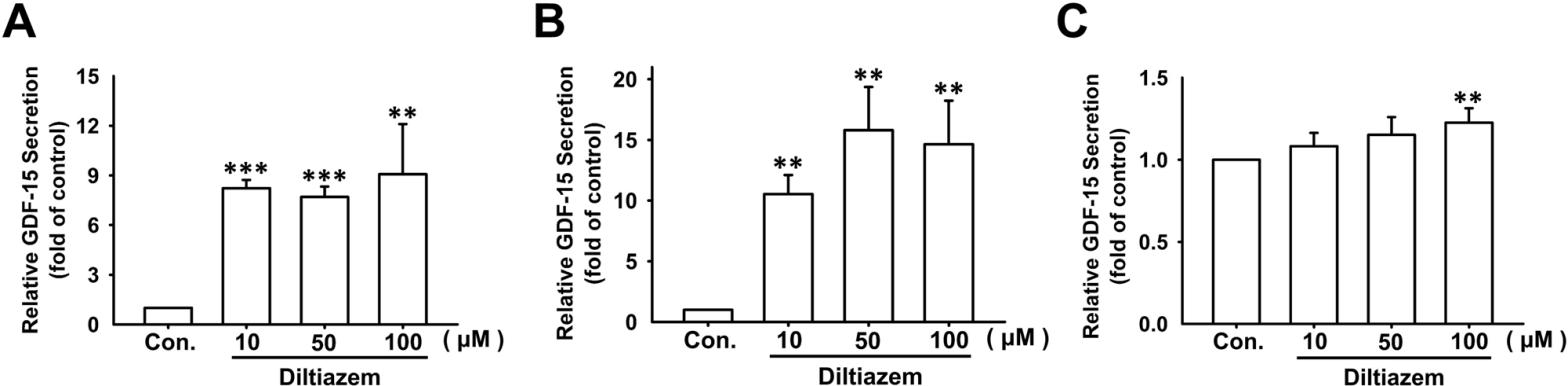
Diltiazem dose-dependently increases GDF-15 protein secretion in breast cancer cell lines. After diltiazem treatment for 24 h, culture media were collected and GDF-15 expression was examined by ELISA. Note that Diltiazem elevated GDF-15 secretion on JC (**A**), 4T1 (**B**) and MDA-MB-231 (**C**) cells. Graphs showed mean ± S.D. of three independent experiments. *p* value was calculated using Student’s *t* test. **p < 0.01; ***p < 0.001 compared to control group.

**Fig. 5:**
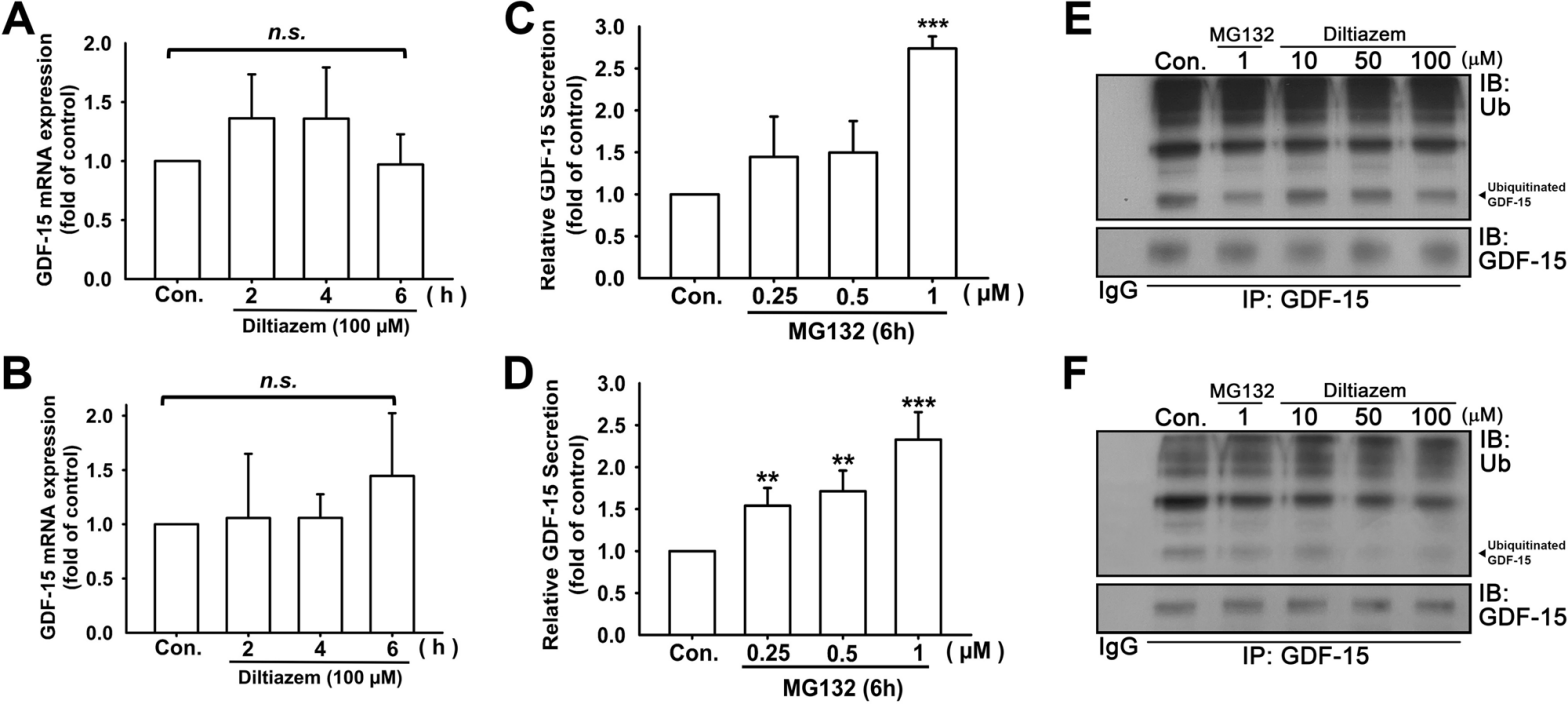
Diltiazem decreases GDF-15 proteasomal degradation in breast cancer cell lines. After treating diltiazem for 2 to 6 h, the mRNA expression of GDF-15 had no significant changes on 4T1 cells (**A**) and MDA-MB-231 cells (**B**) analyzed by real-time PCR. In addition, treating cells with proteasome inhibitor MG132 (0.25–1 μM) for 6 h markedly enhanced GDF-15 secretion in culture media on both 4T1 (**C**) and MDA-MB-231 cells (**D**) examined by ELISA. (**E**, **F**) The cell lysates were immunoprecipitated with normal IgG or anti-GDF-15 antibody. The immunoprecipitated proteins were subjected to SDS-PAGE and immunoblotted against ubiquitin (Ub) and GDF-15. Diltiazem dose-dependently decreased the level of ubiquitinated GDF-15, as well as MG132 did. Graphs showed mean ± S.D. of three independent experiments. *p* value was calculated using Student’s *t* test. **p < 0.01; ***p < 0.001 compared to control group.

GDF-15 is a critical regulator involved in cancer progression and migration. In addition to the regulation of EMT and cell motility by diltiazem, the expression of GDF-15 was significantly enhanced by diltiazem dose-dependently measured by ELISA (Fig. 4). However, as shown in Fig. 5A, B, the mRNA expression of GDF-15 was not significantly elevated in accordance with protein expression. This phenomenon led us to the investigation of the regulation of GDF-15 degradation. After treating cells with MG132, the proteasome inhibitor, the protein expression of GDF-15 analyzed by ELISA was elevated in a dose-dependent manner (Fig. 5C, D). Furthermore, proteins were immunoprecipitated with antibody against GDF-15, and the precipitated proteins were immunoblotted with antibody against ubiquitin. As shown in Fig. 5E, F, treatment of MG132 or diltiazem reduced ubiquitinated GDF-15 levels. These findings suggest that diltiazem upregulates GDF-15 expression at least in part by inhibiting its proteasomal degradation.

### GDF-15 decreases cell motility and epithelial-mesenchymal transition in breast cancer cells

**Fig. 6:**
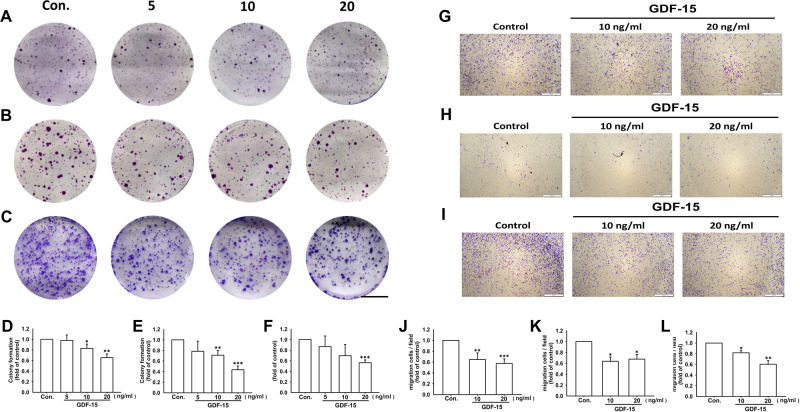
Colony formation and cell migration are inhibited by recombinant GDF-15 in breast cancer cell lines. Both JC and 4T1 cells were treated with murine recombinant GDF-15 protein (5–20 ng/ml), and MDA-MB-231 cells were treated with human recombinant GDF-15 protein (5–20 ng/ml). After GDF-15 treatment for 10 days, the number of colonies formed (bigger than 1 mm in diameter) were counted. Note that colony formation of JC (**A**, **D**), 4T1 (**B**, **E**), and MDA-MB-231 cells (**C**, **F**) were all inhibited by GDF-15. Scale bar, 1 cm. In cell culture insert system, GDF-15 treatment for 24 h also reduced cell migration in JC (**G**, **J**), 4T1 (**H**, **K**), and MDA-MB-231 cells (**I**, **L**). Scale bar, 400 μm. Graphs showed mean ± S.D. of three independent experiments. *p* value was calculated using Student’s *t* test. *p < 0.05; **p < 0.01; ***p < 0.001 compared to control group.

In order to elucidate whether diltiazem-inhibited cell motility and EMT were regulated via GDF-15, recombinant GDF-15 was administered to manifest the regulatory mechanism of diltiazem. As shown in Fig. 6, colonies formed of JC (Fig. 6A, D), 4T1 (Fig. 6B, E) and MDA-MB-231 cells (Fig. 6C, F) were significantly diminished in a dose-dependent manner by GDF-15 administration. Colony formation was reduced to 0.65 ± 0.07-fold, 0.44 ± 0.07-fold, and 0.56 ± 0.05-fold of control under 20 ng/ml GDF-15 treatment on JC, 4T1, and MDA-MB-231 cells, respectively. In cell culture insert system, treatment of recombinant GDF-15 for 24 h markedly decreased cell migration through transwell membrane compared with vehicle-treated control group in JC (Fig. 6G, J), 4T1 (Fig. 6H, K) and MDA-MB-231 cells (Fig. 6I, L). Cell migration was reduced to 0.57 ± 0.09-fold, 0.68 ± 0.15-fold, and 0.57 ± 0.12-fold of control under 20 ng/ml GDF-15 treatment on JC, 4T1, and MDA-MB-231 cells, respectively. These findings suggest that colony formation and cell migration ability were inhibited by GDF-15 in triple-negative breast cancer cells.

**Fig. 7:**
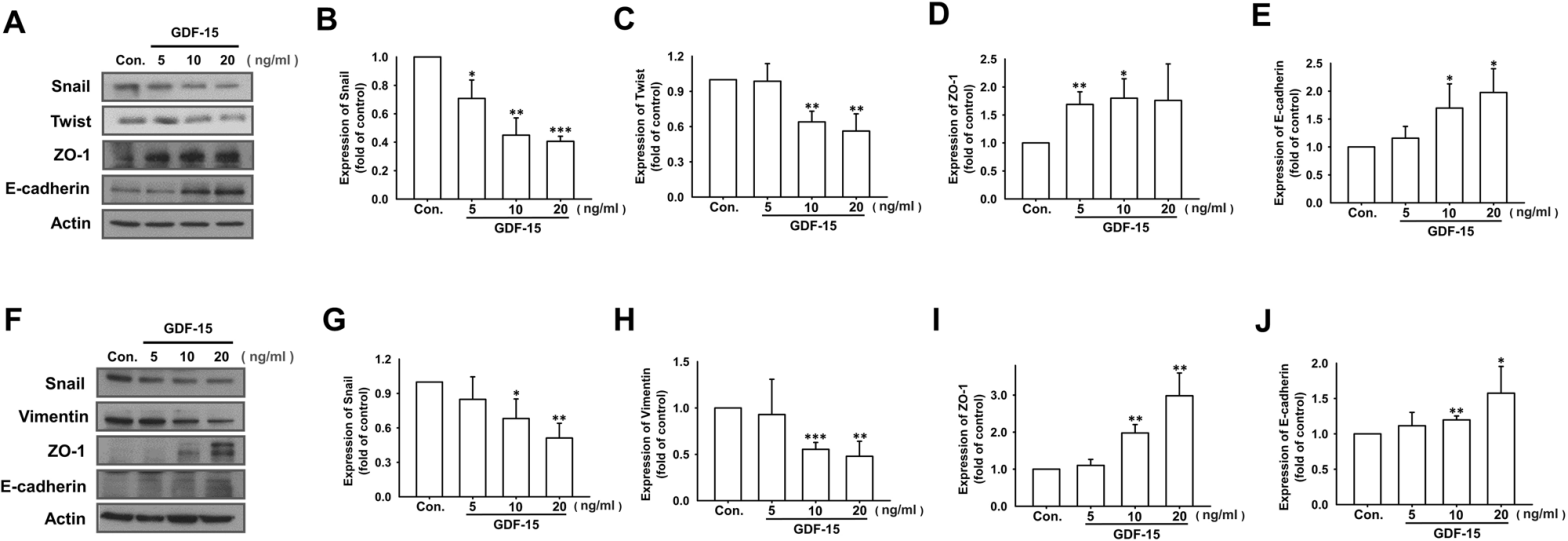
GDF-15 reverses epithelial-mesenchymal transition by increasing epithelial markers expression and decreasing mesenchymal markers expression in breast cancer cell lines. 4T1 cells were treated with murine recombinant GDF-15 protein (5 to 20 ng/ml), and MDA-MB-231 cells were treated with human recombinant GDF-15 protein (5 to 20 ng/ml). After treating with GDF-15 recombinant protein for 24 h, protein expression of epithelial/mesenchymal markers were detected by Western blot. It is shown that recombinant GDF-15 decreased the expression of mesenchymal markers (Snail, Twist and vimentin) but increased the expression of epithelial markers (E-cadherin and ZO-1) on 4T1 **(A**–**E)** and MDA-MB-231 **(F**–**J)** breast cancer cells. Graphs showed mean ± S.D. of three independent experiments. *p* value was calculated using Student’s *t* test. *p < 0.05; **p < 0.01; ***p < 0.001 compared to control group.

Moreover, after treating by GDF-15 for 24 h, protein expressions of Snail, Twist, and vimentin representing as mesenchymal markers were decreased in both 4T1 (Fig. 7A–C) and MDA-MB-231 cells (Fig. 7F–H). On the other hand, protein expressions of epithelial markers including zonula occludens-1 (ZO-1) and E-cadherin were significantly elevated by GDF-15 in both 4T1 (Fig. 7A, D, E) and MDA-MB-231 cells (Fig. 7F, I, J). Taken together, these findings indicate that GDF-15 reverses EMT and inhibit breast cancer motility.

### Diltiazem attenuates pulmonary metastasis in vivo

**Fig. 8:**
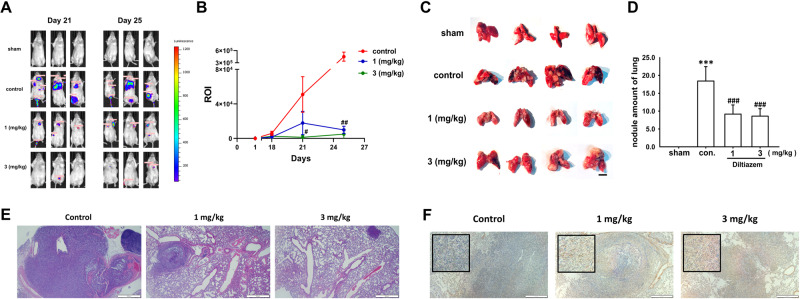
Diltiazem attenuates 4T1 cells colonization to lung in vivo. **A** Representative IVIS images of BALB/c mice 21 days after 4T1-luc cells injection. Diltiazem (1, 3 mg/kg) was given by oral gavage, and ddH_2_O was served as vehicle control. **B** Quantification of luciferase intensity of mice on days 18, 21, and 25. Representative images of dissected lungs and the number of nodules was counted (**C**, **D**). Scale bar, 1 cm. Representative images of H&E staining (**E**) and GDF-15 expression (**F**) of pulmonary slices. Scale bar, 500 μm. Graphs showed mean ± S.D. of at least four independent experiments. *p* value was calculated using Student’s *t* test. ***p < 0.001 compared to sham group. ^###^*p* < 0.001 compared to control group.

4T1-luc breast cancer cells were intravenously injected into female BALB/c mice through tail vein. As shown in Fig. 8A, B, luminescence intensity of 4T1 cells obtained from IVIS system on the 18th, 21st and 25th days demonstrated that diltiazem-treated mice had minor lung metastasis compared with control group. The presence of multiple nodules indicates pulmonary metastasis of 4T1 breast cancers cells, and the treatment of diltiazem significantly reduced the number of pulmonary nodules compared with control group (Fig. 8C, D). Representative images of H&E staining of lung sections showed the density and the size of nodules in Fig. 8E. In addition, Fig. 8F also demonstrated that diltiazem enhanced GDF-15 expression or secretion exhibited by immunohistochemical images.

**Fig. 9:**
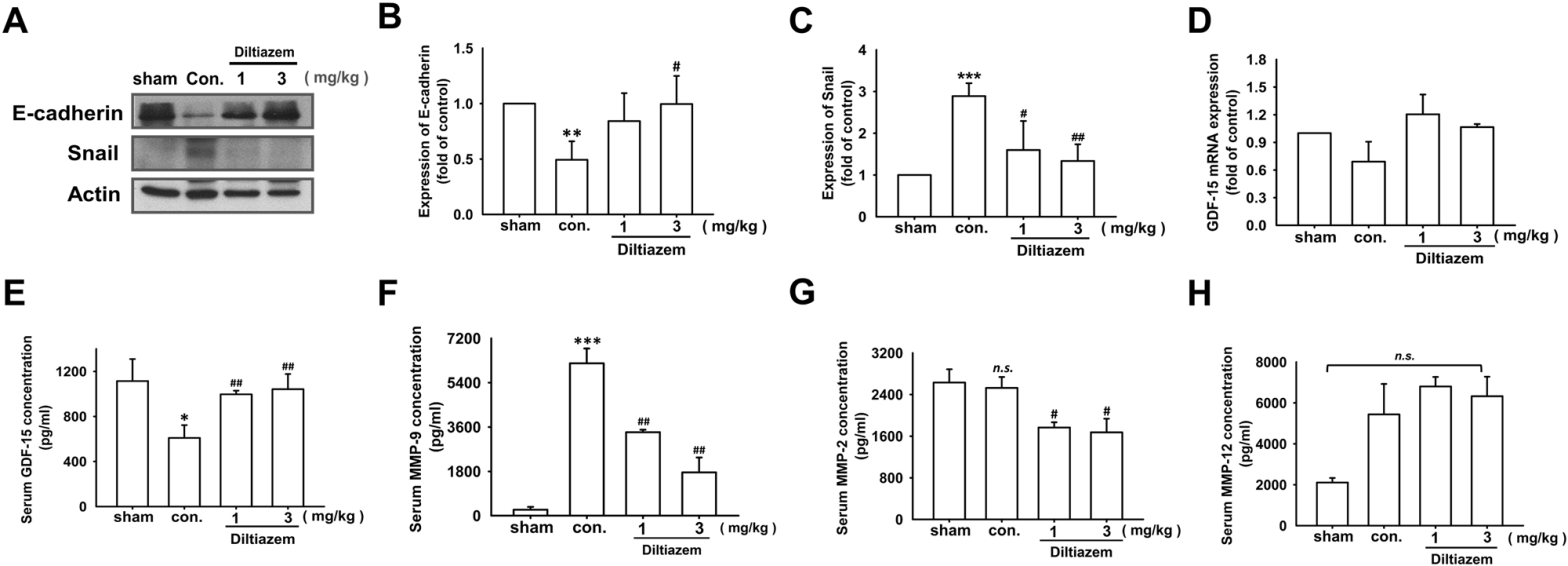
Diltiazem influences EMT markers, GDF-15 and MMPs expressions in vivo. **A**–**D** The protein expression of epithelial marker E-cadherin, mesenchymal marker Snail, and the mRNA expression of GDF-15 were examined by using homogenized lung tissues. Graphs showed mean ± S.D. of at least three independent experiments. **E**–**H** The expression of serum GDF-15, MMP-9, MMP-2, and MMP-12 were measured by ELISA. Graphs showed mean ± S.E.M. of at least four independent experiments. *p* value was calculated using Student’s *t* test. *p < 0.05; **p < 0.01; ***p < 0.001 compared to sham group. ^#^*p* < 0.05; ^##^*p* < 0.01 compared to control group.

Furthermore, by homogenized lung tissues, we found that epithelial markers E-cadherin was increased in diltiazem-treated group, and mesenchymal markers Snail was decreased by diltiazem compared with control group (Fig. 9A–C). The mRNA expression of GDF-15 was not significantly altered, however, serum concentration of GDF-15 was markedly enhanced in diltiazem-treated group compared with control (Fig. 9D, E). In addition, serum concentration of metalloproteinase (MMP)-9 and MMP-2 were both declined by diltiazem, however, no significant difference was observed in MMP-12 expression (Fig. 9F–H). These findings indicate that diltiazem enhances GDF-15 expression, reverses EMT, and reduces MMP-9 and MMP-2 expressions, leading to attenuation of breast cancer lung metastasis.

## Discussion

Calcium as a second messenger regulates cardiac system, metabolism, and various cellular processes. In oncology researches, Ca^2+^ signaling is involved in apoptosis, autophagy and cell cycle [26]. Nevertheless, the relationship between the use of calcium channel blockers (CCBs) and the risk of cancers is a controversial issue for years. In 1990s, it was hypothesized that regular use of CCBs increases the risk of various cancers [27–30]. It was proposed that calcium antagonism leads to apoptosis inhibition resulting in tumor-promoting effect [31, 32]. Several studies tracked and investigated the risk of cancers on patients who were on long-term medication of CCBs. They found some CCBs such as verapamil and nifedipine increased the risks in lung, colorectal, kidney and breast cancers [29, 33]. However, from recent large population-based cohort study, strong evidence has provided that CCBs use is not associated with an increased risk of cancer [34, 35]. Several studies reported that voltage gated Ca^2+^ channels (VGCC) are highly expressed in cancer cells to increase cell survival, thus, inhibition of VGCCs induces autophagy, as well as reduces the Ca^2+^ influx and dynamics to promote apoptosis [26, 36]. Moreover, accumulating studies proved the anti-cancer effects, including inhibition of cell proliferation as well as solving the drug resistance in chemotherapy [21–23, 37]. In this present study, we also confirmed the anti-tumor effects of VGCC diltiazem on triple-negative breast cancers, including inhibition of EMT and cell motility in vitro and in vivo.

Diltiazem, an FDA-approved calcium channel blocker, is indicated for angina, hypertension and congestive heart failure in clinical application. The usual doses for hypertension and angina in adults are among 120 and 180 mg daily, and the maximum dose is 540 mg per day. The general oral dosage for cardiovascular and retinal studies are between 25 and 50 mg/kg per day in experimental mouse model [38–40]. In our studies, dosages ranged from 1 to 3 mg/kg minimize the impact to blood pressure and cardio functions.

Intracellular calcium levels and/or calcium-dependent pathways are factors influencing ubiquitin-proteasome system. It has been reported that calcium activates Nedd4 E3 ligase activity resulting in increased poly-ubiquitin products [41]. Calcium binding domains are presence on several members of the Nedd4 family and CBL family of E3 ligases and exhibit putative calcium binding property [42]. It has been demonstrated that ubiquitination of proteins is observed when intracellular calcium level elevated, and ubiquitination of proteins is suppressed when the entry of calcium ion into the cells is inhibited, suggesting the ubiquitin-proteasome system is activated in response to the elevation of intracellular calcium level [43]. In accordance with our finding, we found that ubiquitination of GDF-15 was suppressed under the treatment of calcium channel blocker diltiazem (Fig. 5), which in part leading to increased GDF-15 secretion.

GDF-15 regulates and involves in a variety of cellular functions, including cardiovascular, metabolism, and kidney diseases [44]. Although being a member of TGF-β superfamily, more and more evidence showed that GDF-15 has high affinity with GDNF family receptor α-like (GFRAL) [45, 46], which is correlated to anti-obesity effects and energy balance [45, 47]. However, the role of GDF-15 in cancers is ambiguous by exerting pro-tumorigenic and anti-tumorigenic functions [48]. Several studies referred that GDF-15 promotes cancer proliferation and metastasis in colorectal [49], cervical [50], pancreatic [51] and lung cancers [52]. Other reports indicates that GDF-15 promotes migration, invasion and cell growth through TGF-β/Smad signaling [49, 50]. On the contrary, evidence indicates that GDF-15 acts as a tumor suppressor by inducing p53-mediated apoptosis in colorectal [53], prostate [54] and breast cancers [55]. It has also been reported that GDF-15 may be decreased under pro-metastatic condition [55]. This is similar with our finding in Fig. 9E showing the serum concentration of GDF-15 was significantly repressed in control tumor-bearing mice compared with sham mice. Wang *et al*. has revealed that decreased GDF-15 expression enhances EMT, cell migration ability in breast cancer cell line, and metastatic nodules in breast cancer-bearing mice [55]. Furthermore, overexpression of GDF-15 diminishes migration ability on both normal mammary gland epithelial cells and breast cancer cells [55]. Enhanced GDF-15 expression also reduces the number and sites of local and distal metastases in vivo compared to control group in lung adenocarcinoma [56]. It has also been reported that anti-cancer drugs, such as Phortress, enhances GDF-15 expression and tumor recession in breast cancers [57]. Consistent with previous studies, we found that serum GDF-15 expression is decreased in breast cancer-bearing mice, and diltiazem-induced GDF-15 exhibits anti-tumor effects by decreasing EMT and metastatic nodules of breast cancer.

In our study, cellular response to diltiazem-induced GDF-15 secretion and the anti-tumor effects of GDF-15 were different in strength in different cell lines. Some possible explanations for the phenomena include: (1) the expression of GDF-15 in different cells lines or species, and (2) the contribution of GDF-15 receptors in different cells. In fact, among TGF-β superfamily members, GDF-15 shows the lowest sequence conservation across species [58]. While sequence of TGF-β1 are 99–100% identical between rat, mouse and human, GDF-15 is below 70%. In addition, the promotor regions are entirely different between humans and mice [59]. Other than that, the receptor for GDF-15 was unknown until recent years. Glial cell-derived neurotrophic factor family receptor α-like (GFRAL) was describes as the sole receptor for GDF-15 [45, 60], which is distantly related to TGF-β receptor family and was originally considered expressed in human brain stem exclusively [61, 62]. With increasing evidence verifying the expression of GFRAL is also in cancer tissues, clues of GFRAL-independent effects of GDF-15 are also accumulating [63–65]. Nevertheless, further investigations are needed to elucidate these points.

## Conclusions

In conclusion, diltiazem attenuates colony formation, cell migration, and EMT by increasing GDF-15 expression level through inhibiting its proteolytic degradation in different breast cancer cell lines in vitro. Diltiazem administration in vivo also upregulates serum level of GDF-15, diminishes EMT and MMP-9/MMP-2 expression, leading to the decrease of lung metastasis of breast cancer.

## Methods & materials

### Materials

Materials used were listed in supplementary file (Supplementary Table 1).

### Cell culture

JC cells, a murine primary breast cancer cell line characterized as basal-like or luminal B type [66], was purchased from Bioresource Collection and Research Center (Hsinchu, Taiwan) and cultured in the RPMI-1640 Medium (Thermo Fisher Scientific, Waltham, MA, USA) supplemented with 1 mM sodium pyruvate and 4.5 g/L glucose. 4T1 cells, a murine triple-negative breast cancer cell line, was provided from American-Type Culture Collection (Manassas, VA) and maintained in RPMI-1640 Medium. MDA-MB-231 cells, human triple-negative breast cancer cell line, was obtained from Bioresource Collection and Research Center (Hsinchu, Taiwan) and have been authenticated by STR profiling. MDA-MB-231 cells were maintained in RPMI-1640 Medium. 10% fetal bovine serum (FBS) and 1% penicillin/streptomycin were added. Cells were cultured in 37 °C incubator with 95% air and 5% CO_2_. All cells used in this study were tested for mycoplasma. Cells that subjected to following assays were seeded in 6-well, 24-well, or 96-well plates, and experimental processes were conducted sequentially as designs shown in figures. No blinding was done.

### Cell viability

To investigate the cytotoxic effects on diltiazem, JC, 4T1 and MDA-MB-231 cells were seeded in 96-well plates with the density of 1 × 10^4^ cells/well and exposed to 0 to 100 µM diltiazem for 24 and 48 h with or without serum. In MTT assay, the cells were incubated with 0.5 mg/ml MTT solution dissolved in cultured medium in 37 °C incubator for an hour. After washing, cells were lysed with DMSO, and measured by SpectraMax M5 plate reader (Molecular Devices, Sunnyvale, CA, United States) with O.D. 550 nm. In proliferation assay, cells were photographed after indicated treatment and the numbers of cells were counted.

### Colony formation

1 × 10^3^ cells/well were seeded into 6-well plates and maintained in culture medium with 10% serum. Indicated treatment was given in the following day. Medium mixed with diltiazem (0~100 μM) or recombinant GDF-15 protein (0~20 ng/ml) were refreshed every 3 days. At the end of the treatment (the 10th day), cells were stained with 1% crystal violet solution for 5 min, and the number of colonies was counted by ImageJ, with diameter larger than 1 mm [67].

### Cell migration and invasion

We investigated cell migration ability by cell culture insert system. By using transwell, cells were resuspended in medium containing 10% FBS, and 2 × 10^4^ cells were seeded in the upper chamber of cell culture inserts. 600 μL medium containing 15% FBS was added in the lower chambers (24-well, 8-μm pore size, Costar New York, NY, USA). After 6 h for letting adhere, 50 μL medium mixed with diltiazem (0, 50 or 100 μM) or recombinant GDF-15 protein (0, 10 or 20 ng/ml) were added into the upper chambers. After 24 h treatment, medium was gently removed and stained with 0.1% crystal violet for 5 min. The chambers were then washed by PBS twice to removed excess crystal violet. Cells in the upper chambers were scraped off carefully by cotton swabs before being photographed. In invasion assay, 4 × 10^4^ cell were seeded in the upper chamber of cell culture inserts which were coated with 50 µl/cm^2^ Corning Matrigel Basement Membrane Matrix (Product number: 354234).

### Wound healing assay

2-well silicone culture inserts were first placed in 24-well plates. 5 × 10^4^ cells suspended in 60 μl culture medium were seeded in each well. After letting adhere for 24 h, inserts were removed carefully and the cell-free gaps were formed. Meanwhile, 500 μl serum free medium was added into 24-well plates, and the photos for 0-h timepoint were taken under the microscope. After adding another 500 μl serum free medium with or without indicated treatment for another 24 h, the photos for 24-h timepoint were then taken.

### Immunoprecipitation and western blot analysis

After indicated treatment in a 6-well plate format, cells were washed by PBS and lysed by RIPA buffer (150 mM NaCl, 50 mM Tris-HCl, pH 7.4, 1 mM EGTA, 1% NP-40, 0.25% deoxycholate and protease inhibitors cocktail). Samples were votexed and placed on ice for 20 min prior to centrifuging 13,500 rpm for 25 min. Next, supernatant was collected and quantified by using the pierce™ BCA protein analysis kit (Thermo Scientific, Waltham, MA, USA). For immunoprecipitation, 500 µg protein lysates were incubated with 20 µl Protein G Mag Sepharose^®^ Xtra (Cytiva, Marlborough, MA, US) along with 20 µg anti-GDF-15 antibody at 4 °C overnight. The Protein G Mag Sepharose was then washed by elution buffer 3 times, and 2X sample buffer was added prior to being heated at 100 °C for 10 min. Whole cell lysates or immunoprecipitates were then seperated in 8% ~12% SDS-PAGE and transferred to PVDF membrane (Millipore, Billerica, MA, USA). After blocking with 7.5% skim milk dissolved in TBST for 2 h, membranes were hybridized with primary antibodies overnight. On the second day, membranes were washed by TBST thrice and hybridized with secondary anti-mouse or anti-rabbit antibodies under room temperature for 1 h. Protein signals were excited by using enhanced chemiluminescence (EMD Millipore, Billerica, MA, USA) and exposed to Fujifilm Super RX-N films (Valhalla, NY, USA). The protein expression was then quantified by ImageJ.

### Enzyme-linked immunosorbent assay (ELISA)

Cultural supernatant was collected for measuring GDF-15 secretion from 1 × 10^6^ cells per well in 6-well plates by using human and murine GDF-15 ELISA kit. Serum of the mice was collected for measuring GDF-15, MMP-9, MMP-2, and MMP-12 expression by murine ELISA kit accordingly. The procedures were conducted by following the standard protocols provided by manufactorers.

### Quantitative real-time PCR

Cells in 6-well plates were lysed by using TRIzol Reagent (Thermo Fisher Scientific, Waltham, MA) after indicated treatment. Total RNA was extracted and quantified by BioDrop spectrophotometer (BioDrop Ltd., Cambridge, UK). 2 μg RNA was then reverse transcribed into cDNA using cDNA Reverse Transcription Kit (Thermo Fisher Scientific, Waltham, MA). Primers mixed with SYBR Green Master Mix (Applied Biosystem, Singapore) and cDNA samples were both loaded into MicroAmp™ Optical 96-Well Reaction Plate (Applied Biosystem, Singapore). The reacting condition was set as 95 °C for 5 min, 45 cycles at 95 °C for 10 s, and 60 °C for 1 min. The sequence of primers used are provided in Supplementary Table 1.

### Immunofluorescence

To investigate actin dynamics in breast cancer cell lines, FITC-phalloidin Reagent (Abcam, Waltham, MA) was used to stain F-actin. 5 × 10^4^ cells were seeded in 24-well plates with glass coverslips inside, and medium containing 50 μM diltiazem was added after cell attachment. After another 24 h, medium was removed. Cells were fixed by 4% formaldehyde for 15 min and permeabilized by 0.1% triton x-100 for 5 min. 5% skim milk dissolved in PBS were added and blocked for 1 h. The coverslips were then incubated with 1X phalloidin reagent and placed in a dark place for 75 min. Before mounting the coverslips, cells were incubated with DAPI (1 μg/ml) for 5 min. We washed the cells with PBS thrice before every steps. Cells were then photographed under fluorescence microscope (Olympus, Tokyo, JP) at 360 nm and 495 nm wavelength.

### Mouse tumor-bearing models

All experiments were approved by Institutional Animal Care and Use Committee (CMUIACUC-2020-318, China Medical University, Taichung, Taiwan). 7-week-old female BALB/c mice (19 ± 1 g) were purchased from National Laboratory Animal Center (Taipei, Taiwan) and housed in laboratory animal center (China Medical University, Taichung, Taiwan) with humidity and temperature controled. Diet and water were under free access. After adapting for a week, 200 μL serum-free medium containing 1 × 10^5^ 4T1-luc cells (labeled with luciferase) were given by tail intravenous injection while the sham group was injected with 200 μL serum-free medium with no cancer cells. After injection, cancer-bearing mice were randomly grouped before diltiazem (1 or 3 mg/kg in saline) or saline was administered by oral gavage once a day, 5 days a week. There were 4 mice in sham group, and 6 mice in each experimental group. Body weight and blood pressure had been monitored, and mice were sacrificed on the 30th day. There were 2 mice in control group died before designed end point, and the data collected from these 2 mice were exluded from analysis. After deep anesthesia, cardiac puncture blood collection and organ (lung, spleen and liver) disection were performed. Post-caval lobe lung tissues were lysed by RIPA buffer or TRIzol using Precellys 24 Tissue Homogenizer (Bertin Instrument, France). No blinding was done.

### In vivo imaging

To trace luciferase labled-4T1 breast cancer cells in live mice, mice were injected intra-peritoneally with D-luciferin (10 μL/g, dissolved in DPBS, AAT Bioquest, California) 10 min before anesthesia using 2.5% isoflurane by veterinary anesthesia vaporizer. Luminescence intensity was then detected and analyzed by IVIS Lumina LT Series III (PerkinElmer, Waltham, MA).

### Immunohistochemistry

Disected lungs were subjected to 10% formalin fixation followed by paraffin embedment. Lung slices were stained with hematoxyline and eosin (H&E) or hybrisdized with antibodyes. In breif, lung slices were first rehydrated and treated with Hydrogen Peroxide Block (ThermoFisher) and then Protein Block (ThermoFisher). After blocking, the slices were incubated with primary antibody against GDF-15 (Sigma-Aldrich) overnight. The slices were then subjected to secondary Antibody Enhancer and Polymer-HRP (GBI LABS), followed by using diaminobenzene (ThermoFisher) as the chromogen before being mounted.

### Statistics

Statistical analysis was performed by using GraphPad Prism and SigmaPlot softwares. Values are expressed as mean ± S.D. of three independent experiments unless otherwise stated. Sample sizes are calculated for significance to be reached. No pre-test was performed to choose sample size. No data points was exluded. Results were analyzed with Student’s *t*-test, and significance was defined as *p* < 0.05.

## Supplementary information

Supplementary Materials

## Data Availability

The datasets used and/or analyzed during the current study are available from the corresponding author on reasonable request.
