# Supplementary material for: Diltiazem inhibits breast cancer metastasis via mediating growth differentiation factor 15 and epithelial-mesenchymal transition
Source: Oncogenesis. 2022 Aug 13;11(1):48. doi: 10.1038/s41389-022-00423-5 (PMC9376069; doi:10.1038/s41389-022-00423-5)
Supplement: Supplementary file 1 — Supplementary Materials [file 41389_2022_423_MOESM1_ESM.docx]

| **Materials** | **Brand (Catalog Number)** |
| --- | --- |
| Diltiazem | Abcam (ab120260), reconstitute in water |
| Recombinant Mouse GDF-15 | Novus (NBP2-51921) |
| Recombinant Human GDF-15 | Peprotech (120-28C) |
| E-cadherin antibody | Cell Signaling (# 3195) |
| ZO-1 antibody | Invitrogen (# 61-7300) |
| Vimentin antibody | Santa Cruz (sc-6260) |
| Snail antibody | Cell Signaling (# 3879) |
| Twist antibody | Santa Cruz (sc-81417) |
| GDF-15 antibody for WB | Abcam (ab105738) |
| GDF-15 antibody for IP | Santa Cruz (sc-515675) |
| GDF-15 antibody for IHC | Sigma-Aldrich (SAB5700031) |
| Ubiquitin | Santa Cruz (sc-8017) |
| α-Tubulin antibody | Sigma-Aldrich (T5168) |
| β-Actin antibody | Sigma-Aldrich (A5441) |
| GAPDH antibody | BioLegend (649202) |
| Anti-mouse antibody | Sigma-Aldrich (AP124P) |
| Anti-rabbit antibody | Sigma-Aldrich (AP132P) |
| Mouse MMP9 ELISA kit | Abcam (ab253227) |
| Mouse MMP2 ELISA kit | Abcam (ab254516) |
| Mouse MMP12 ELISA kit | Abcam (ab246540) |
| Mouse GDF15 ELISA kit | Abcam (ab216947) |
| Human GDF15 ELISA kit | Abcam (ab155432) |
| Phalloidin FITC Reagent | Abcam (ab235137) |
|  |  |
| **Primers** | **Sequences** |
| Mouse GDF-15 | F: GAGCTACGGGGTCGCTTC  R: GGGACCCCAATCTCACCT |
| Human GDF-15 | F: TGCCCGCCAGCTACAATC  R: TCTTTGGCTAACAAGTCATCATAGGT |
| Mouse 36B4 | F: AGATGCAGCAGATCCGCAT  R: GTTCTTGCCCATCAGCACC |
| Human 36B4 | F: TGACGGGGTCACCCACACTGTGCCCATCTA  R: CTAGAAGCATTTGCGGTGGACGATGGAGGG |
| Human β-Actin | F: TGACGGGGTCACCCACACTGTGCCCATCTA  R: CTAGAAGCATTTGCGGTGGACGATGGAGGG |

**Supplementary Table 1. Materials and primers used in this study.**

**
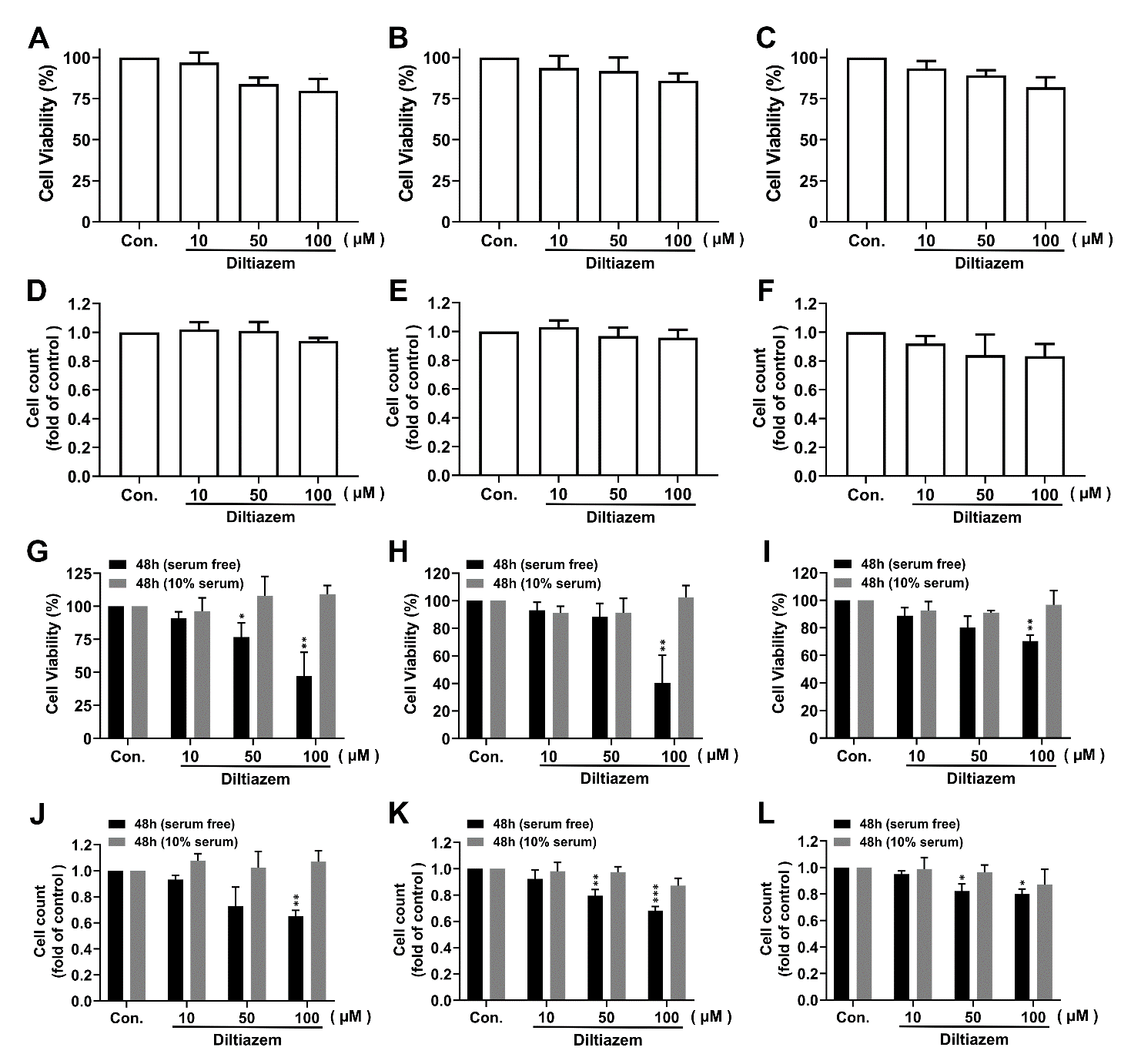
**

**Supplementary Figure 1. Cell viability of breast cancer cells after diltiazem treatment for 24 or 48 hours.** (**A** to **F**) Cells were treated under indicated dosages of diltiazem without serum for 24 hours, and MTT assay (**A** to **C**) and proliferation assay (**D** to **F**) were used to examine cell viability on JC (**A**, **D**), 4T1 (**B**, **E**), and MDA-MB-231 cells (**C**, **F**). (**G** to **L**) Cells were treated under indicated dosages of diltiazem with or without serum for 48 hours, and MTT assay (**G** to **I**) and proliferation assay (**J** to **L**) were used to examine cell viability on JC (**G**, **J**), 4T1 (**H**, **K**), and MDA-MB-231 cells (**I**, **L**). Note that the negative effcts observed under 48 hours’ treatment of diltiazem without serum were not seen with the presence of serum, suggesting the safety of these dosages of diltiazem. Graphs showed mean ± S.D. of three independent experiments. *p* value was calculated using Student’s t test. ^*^*p* < 0.05; ^**^*p* < 0.01; ^***^*p* < 0.001 compared to control group.


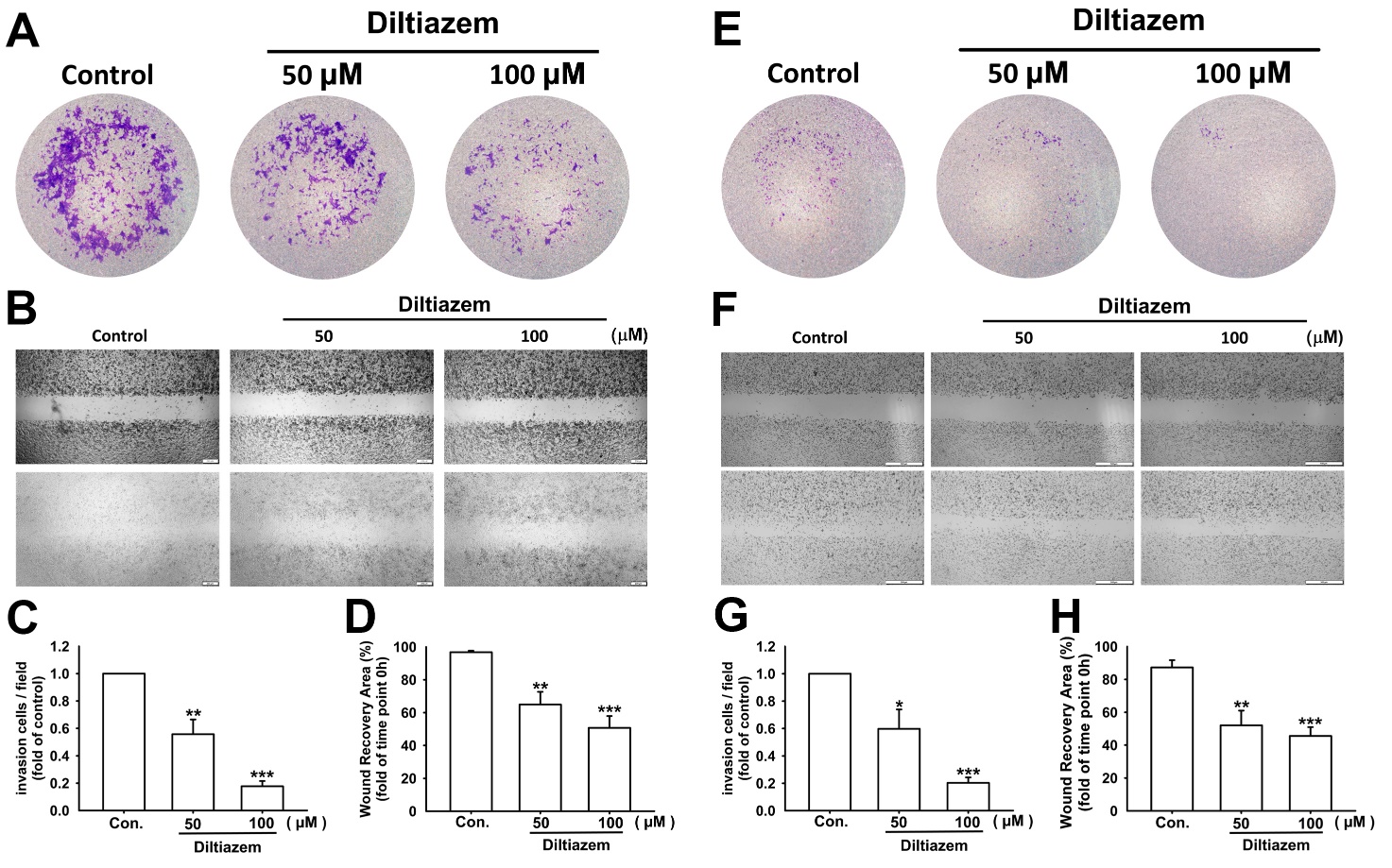


**Supplementary Figure 2. Cell invasion and wound healing ability of breast cancer cells after diltiazem treatment.** Diltiazem treatment for 24 hours reduces cell invasion (**A** and **C**) and wound healing ability (**B** and **D**) in 4T1 cells. Diltiazem treatment for 24 hours also markedly reduces cell invasion (**E** and **G**) and wound healing ability (**F** and **H**) in MDA-MB-231 cells. Graphs showed mean ± S.D. of three independent experiments. *p* value was calculated using Student’s t test. ^*^*p* < 0.05; ^**^*p* < 0.01; ^***^*p* < 0.001 compared to control group.
